# Correlation between *Hox* code and vertebral morphology in the mouse: towards a universal model for Synapsida

**DOI:** 10.1186/s40851-017-0069-4

**Published:** 2017-06-13

**Authors:** Christine Böhmer

**Affiliations:** UMR 7179 CNRS/MNHN, Muséum National d’Histoire Naturelle, 57 rue Cuvier CP-55, Paris, France

**Keywords:** Axial skeleton, Evolution, Mammalia, Regulatory genes, Phenotypic variation

## Abstract

**Background:**

The importance of the cervical vertebrae as part of the skull–neck system in facilitating the success and diversity of tetrapods is clear. The reconstruction of its evolution, however, is problematic because of the variation in the number of vertebrae, making it difficult to identify homologous elements. Quantification of the morphological differentiation in the neck of diverse archosaurs established homologous units of vertebrae (i.e. modules) resulting from *Hox* gene expression patterns within the cervical vertebral column. The present study aims to investigate the modularity of the cervical vertebral column in the mouse and to reveal the genetic patterns and changes underlying the evolution of the neck of modern mammals and their extinct relatives. In contrast to modern mammals, non-mammalian synapsids are characterized by a variable cervical count, the presence of free cervical ribs and the presence of a separate CV1 centrum. How might these evolutionary modifications be associated with changes in the *Hox* code?

**Results:**

In combination with up-to-date information on cervical *Hox* gene expression including description of the vertebral phenotype of *Hox* knock-out mutants, the 3D landmark-based geometric morphometric approach demonstrates a correlation between *Hox* code and vertebral morphology in the mouse. There is evidence that the modularity of the neck of the mouse had already been established in the last common ancestor of mammals, but differed from that of non-mammalian synapsids. The differences that likely occurred during the evolution of synapsids include an anterior shift in *HoxA-5* expression in relation to the reduction of cervical ribs and an anterior shift in *HoxD-4* expression linked to the development of the highly differentiated atlas-axis complex, whereas the remaining *Hox* genes may have displayed a pattern similar to that in mammals on the basis of the high level of conservatism in the axial skeleton of this lineage.

**Conclusion:**

Thus, the mouse *Hox* code provides a model for understanding the evolutionary mechanisms responsible for the great morphological adaptability of the cervical vertebral column in Synapsida. However, more studies in non-model organisms are required to further elucidate the evolutionary role of *Hox* genes in axial patterning of the unique mammalian body plan.

## Background

The evolution of a morphologically distinct and functional neck consisting of a series of cervical vertebrae (CV) had a great impact on the ecological diversification of tetrapods due to its involvement in a number of vital functions [1–3]. These functions most notably include feeding behavior and locomotion, but also sexual display and combat behavior (“necking”) [2–6]. Despite the great numerical and morphological variety in the cervical vertebral column, tetrapods share a common developmental regulatory program that mediates axial patterning during embryogenesis [7–9]. During the process of somitogenesis, morphologically similar segmental units (somites) form in the paraxial mesoderm and subsequently differentiate into morphologically distinct vertebrae (reviewed in [10]). Differentiation of the somites into vertebrae is controlled by the combinatorial expression of *Hox* genes (reviewed in [11]). Evolutionary changes in the vertebral column have been associated with changes in the spatiotemporal patterns of *Hox* gene expression [7–9]. For instance, expression of the *Hox-C6* gene starts at the first thoracic vertebra in a variety of tetrapod species that differ in cervical count [7, 8, 12]. It corresponds to the transition from cervical to thoracic vertebrae (i.e. cervicothoracic transition) in mouse (seven CV), chicken (14 CV), goose (17 CV), crocodile (nine CV) and turtle (eight CV) [7, 8, 12]. Even within the cervical vertebral column of tetrapods, differences in the number of vertebrae and, thus, in the morphological regionalization of the neck correspond to modifications in *Hox* gene expression domains (expansion of a *Hox* gene’s expression domain and/or a shift of gene expression) [13].

In contrast to non-mammalian tetrapods, mammals are highly constrained in the number of cervical vertebrae (almost exclusively seven CV) and their neck kinematics rely on interspecific variation in vertebral morphology, but not in vertebral count [1, 2], Although phylogenetically diverse, there is evidence for a common *Hox* code in living placental mammals, because they appear to display similar patterns of morphological differentiation within the neck, which is thought to reflect a common developmental regionalization [14, 15]. On basis of the high level of conservatism in the axial skeleton of the mammalian lineage, it may be possible that this applies to synapsids in general. Therefore, the first step to test the hypothesis is to analyze the relation between *Hox* code and vertebral morphology in mouse. Johnson and colleagues [16] studied the relation between the change in the number of active *Hox* genes and the 2D shape change between vertebrae in mouse and found a correlation between both. Since then, the *Hox* code is more completely characterized; the present study thus aims to expand on previous work by summarizing up-to-date information on *Hox* gene expression, including information on the vertebral phenotype of *Hox* knock-out mutants (as direct evidence for the relationship between genotype and phenotype). Furthermore, the present work applies a 3D vertebral shape analysis, which was not feasible in the past. This makes it possible to test if the 3D method will show the similar pattern in shape change between vertebrae in the mouse neck as revealed by the 2D study. In a second step, the morphological changes in the axial skeleton and potentially associated genetic modifications during synapsid evolution will be discussed. The differences may be linked with shifts in *Hox* gene expression, whereas similarities may indicate a similar *Hox* gene expression pattern on the basis of the high level of conservatism in the axial skeleton of this lineage. As a result, this will reveal if the morphological variation within the cervical vertebral column of the mouse may serve as a *Hox* gene expression pattern proxy in synapsids in general.

Reconstruction of *Hox* gene expression patterns based on vertebral morphology has only recently become possible [13, 17]. The correlation between anterior *Hox* gene expression and the quantifiable shape of the cervical vertebrae of living archosaurs (crocodile, alligator, and chicken) has shown that changes in the expression of the underlying genetic code can be deduced solely from vertebral morphology [13]. Furthermore, the correlation observed in extant crocodiles and birds allowed the reconstruction of the hypothetical vertebral *Hox* code in an extinct relative, the dinosaur *Plateosaurus*, which lacks preserved DNA and is known only from fossils [13]. Differences in the morphological subunits (modules) within the neck suggested that modifications in the expression of *Hox* genes have occurred during archosaur evolution [8, 13].

Among mammals, the vertebral *Hox* code is solely known for the model species mouse [7, 18] but the presence of the respective *Hox* genes in the genome of other placental mammals, marsupials and monotremes is confirmed [19]. The conservation of *Hox* function across different species as shown by previous analyses [7–9, 20] and the conservative number of cervical vertebrae in mammals (virtually always seven CV) [21–23] suggest that the *Hox* gene expression pattern as seen in the neck of the mouse was already established in the last common ancestor of mammals. Yet, the hypothesis that all living mammals share the identical *Hox* code remains to be tested.

The aim of the present study is to investigate the morphological modularity of the cervical vertebral column in the mouse and to analyze the role of previously published *Hox* gene expression in determining proper vertebral morphology. Next, the correlation between anterior *Hox* gene expression and the quantifiable shape of the cervical vertebrae in mouse is tested. As a result, the observations are discussed in the context of synapsid evolution in order to evaluate if the mouse *Hox* code provides a universal model for Synapsida. In contrast to modern mammals, non-mammalian synapsids are characterized by a variable cervical count, the presence of free cervical ribs and the presence of a separate CV1 centrum. Therefore, the following hypotheses will be tested: (1) Is the fixation of cervical count during evolution related to a mouse-like *Hox* code? (2) Is the absence of free cervical ribs associated with a shift in *HoxA-5* expression? (3) Is the evolutionary fusion of the odontoid process to CV2 linked with a shift in *HoxD-4* expression? Ultimately, this will improve our understanding of the evolutionary mechanisms responsible for the great morphological adaptability of the cervical vertebral column that has contributed to the evolution of the unique mammalian body plan.

## Methods

### Quantitative morphological analysis

Morphological variability within the cervical vertebral column of the mouse is evaluated by a landmark-based geometric morphometric analysis (following the procedure described in [13]). To date, this procedure represents the best possible method for identifying morphological modules in vertebral series comprising less than 15 vertebrae (as compared to the linear regression method described in [17] since the general rule of thumb is a minimum number of at least 10 to 20 observations for a regression analysis [24].

**Fig. 1:**
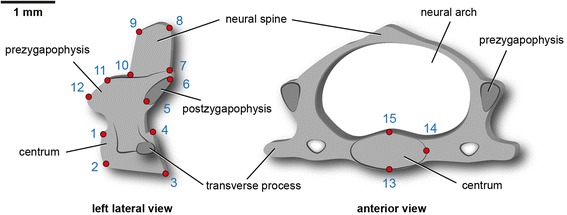
Landmark set used in the 3D geometric morphometric analysis. The numbered 3D landmarks (*red points*) are shown on a schematically illustrated mid-cervical vertebra of *Mus musculus*. Detailed definitions of the 15 homologous points are provided in Table 1

The morphometric approach allows the statistical assessment of shape changes between successive vertebrae. A series of 15 homologous landmarks are digitized on the three-dimensional scans of the cervical vertebrae (CV2 to CV7) using the software LANDMARK v. 3.0 [25] (Fig. 1). The 3D data (specimen TMM M-8671 from the Texas Memorial Museum) is available from the DigiMorph digital library (http://www.digimorph.org/specimens/Mus_musculus/heterozygous/adult/whole/). The homologous points capture the vertebral shape in three dimensions characterizing the morphology of the vertebral centrum and the neural arch (Table 1).

**Table 1:** Definition of landmarks (LM) applied to 3D scans of the mouse vertebrae

LM	Definition
1	dorsal-anterior edge of vertebral centrum
2	ventral-anterior edge of vertebral centrum
3	ventral-posterior edge of vertebral centrum
4	dorsal-posterior edge of vertebral centrum
5	anteriormost edge of articular facet of postzygapophysis
6	dorsal-posterior edge of articular facet of postzygapophysis
7	point of maximum curvature between postzygapophysis and neural spine
8	posterior edge of neural spine
9	anterior edge of neural spine
10	point of maximum curvature between neural spine and prezygapophysis
11	posteriormost point of articular facet of prezygapophysis
12	dorsal–anterior edge of articular facet of prezygapophysis
13	ventralmost point of vertebral centrum
14	lateralmost point of vertebral centrum
15	dorsalmost point of vertebral centrum

The atlas (first cervical vertebra) is not included in the geometric morphometric analysis due to its highly unique morphology. It lacks specific serial homologies with postatlantal cervicals, and thus, several landmarks cannot be applied to it.

Analysis and visualization of the geometric morphometric data is performed using the software MORPHOLOGIKA [26]. First, the 3D coordinates of all landmarks are superimposed using a generalized Procrustes analysis (GPA). The superimposition removes all information unrelated to shape [27]. Next, a relative warps (RW) analysis is performed to reduce the dimensionality of the dataset. With the applied settings, this method is equivalent to a principal components analysis, and reveals similarity relationships among vertebrae within the cervical vertebral column. The RW analysis constructs a morphospace in which shape variation can be quantified. The shape differences are visualized with three-dimensional thin-plate splines.

Furthermore, a cluster analysis using the single linkage algorithm in combination with the Euclidean similarity index is performed on the superimposed landmark coordinates. This joins the vertebrae based on the smallest distance between them.

Eventually, the quantitative morphological analysis results in the establishment of the morphological subunit pattern in the cervical vertebral column.

### *Hox* gene expression and morphological proxies

Generally, the expression of *Hox* genes of the paralog groups (PG) 3 to 6 are involved in mediating the development of the cervical vertebral column (e.g. [28]). In particular, the anterior expression limits of *Hox4* and *Hox5* PG are responsible for the regional patterning in the neck and are the focus of the present study. The somitic *Hox* gene expression pattern in the cervical vertebral column of the mouse is established by a literature survey. The survey focused on embryonic stages at which the somites are developed along the full anteroposterior body axis and the somitic *Hox* gene expression limits are thought to be well established and stable during further development [7–9].

To establish phylogenetic homology [29, 30] between *Hox* gene expression in living archosaurs, the *Hox* gene expression patterns were compared in relation to vertebral morphology in crocodilians and birds [13]. Given the sister-taxon relationship of these two groups, finding the same *Hox* gene expression boundaries coincide with vertebral subunits are most parsimoniously explained as implying homology between these modules [13]. These results from living archosaurs were then used as phylogenetic bracket to hypothesize *Hox* gene expression patterns from vertebral morphology in the most recent common ancestor of birds and crocodilians, and in a fossil representative of this clade [13]. On the basis of the correlation between gene expression and phenotypic changes noted above, the present study tests if the morphological variation of the cervical vertebrae in mouse may serve as a proxy for the mammalian cervical *Hox* code.

Loss-of-function mutations in *Hox4* and *Hox5* PG and their effect on the axial skeleton in the mouse are collected from previously published works.

## Results

### Morphological variation within the cervical vertebral column in mouse

**Fig. 2:**
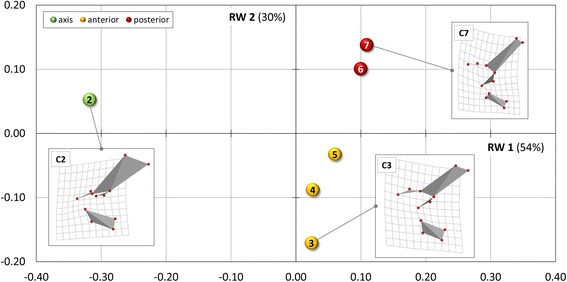
Relative warps (RW) analysis results. The plot shows the shape differences of the cervical vertebrae along RW 1 and RW 2 for *Mus musculus*. Thin-plate splines (3D in left lateral view) visualize the variation between landmark configurations of the vertebrae from the mean shape (zero point). As confirmed by the cluster analysis, the morphological analysis allowed discrimination of the vertebrae in three different subunits (indicated by color coding)

The landmark-based geometric morphometric analysis indicates a distinct morphological differentiation of the neck in the mouse (Fig. 2). About 84% of the total variance in the sample is explained by the first two RWs (Table 2) and, thus, the morphospace constructed from RW 1 and RW 2 provides a reasonable approximation of the total shape variation (Fig. 2). The scatter plot shows that the axis (CV2) is in the second quadrant, the next three cervical vertebrae (CV3-5) are in the fourth quadrant, whereas the last two cervical vertebrae (CV6-7) are in the first quadrant. RW 1 that separates CV2 from the postaxial vertebrae, is largely associated with shape differences of the vertebral centrum, the pre-zygapophyses and the neural spine. RW 2 that separates the anterior group (CV3-5) from the posterior group (CV6-7), is mainly related to shape differences of the vertebral centrum and the post-zygapophyses (Fig. 2).

**Table 2:** Percentage of total variance explained and cumulative variance per relative warp (RW)

RW	Variance [%]	Cumulative variance [%]
1	54.12	54.12
2	29.63	83.75
3	9.10	92.85
4	4.98	97.83
5	2.17	100.00

As confirmed by the cluster analysis, the RW analysis allowed discrimination of the vertebrae in three different subunits including the axis, three anterior and two posterior vertebrae in the mouse.

### *Hox* gene expression in the cervical vertebral column in mouse

**Fig. 3:**
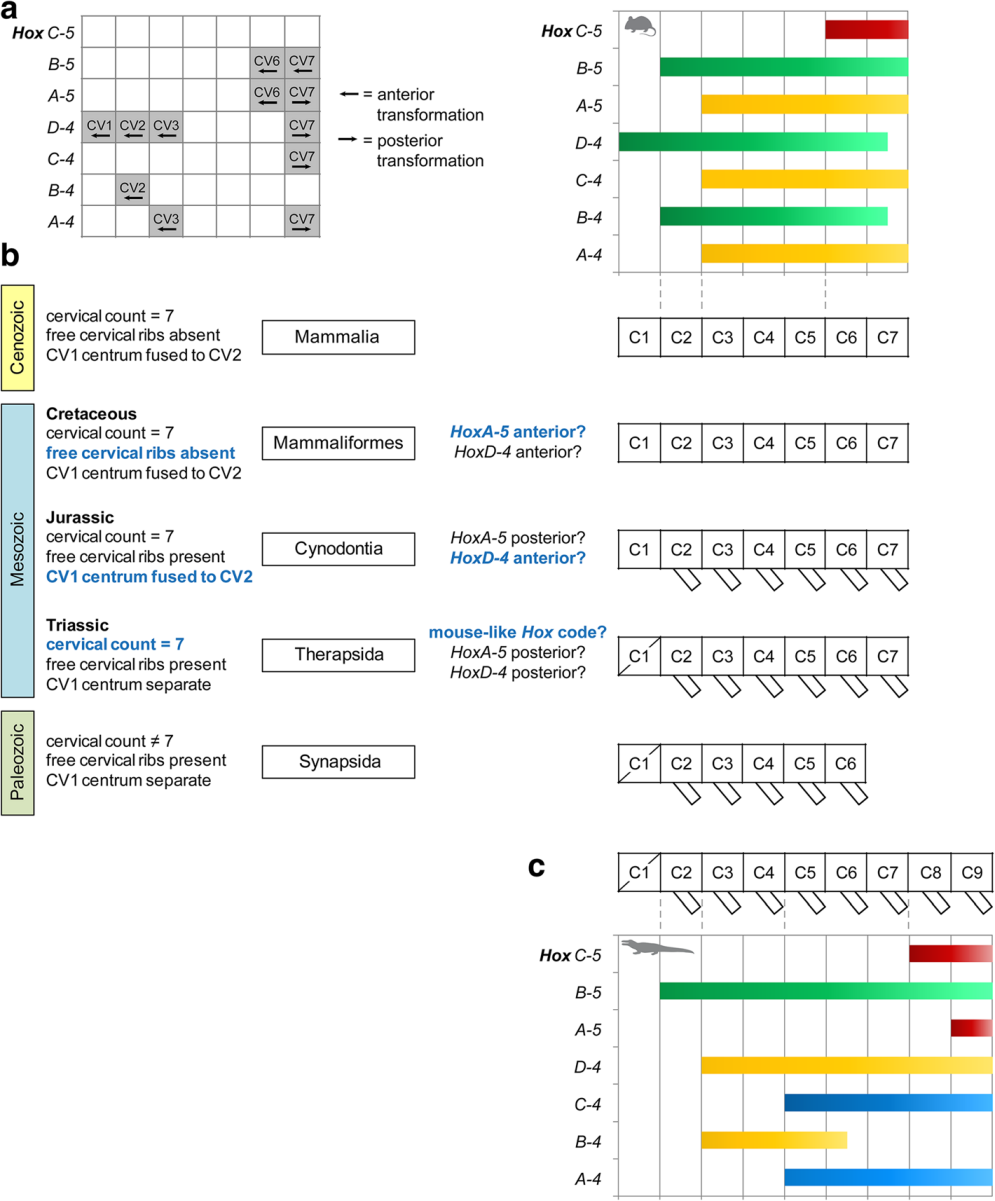
*Hox* code and vertebral morphology through deep time. **a** Effect of single loss-of-function mutations in *Hox4* and *Hox5* paralog group on the cervical vertebral column in the mouse. **b** Overview and schematic illustration of characteristic features in the cervical vertebral column during synapsid evolution. The correlation between somitic *Hox* gene expression pattern and morphological modularity in the neck of *Mus musculus* (top right) allows to hypothesize about the *Hox* code in fossil relatives on basis of major morphological changes. Major morphological and reconstructed genetic changes are indicated in blue. The mouse *Hox* code is based on references provided in Table 3. **c** The correlation between somitic *Hox* gene expression pattern and morphological modularity in the neck of the crocodile serves as outgroup configuration (based on [13]). The crocodilian *Hox* code is based on references [8, 9, 13]

The somitic *Hox* gene expression pattern in the neck of the mouse is summarized in Fig. 3 (see Table 3 for references). The seven *Hox* genes of paralog groups 4 and 5 mediate the specification of the cervical vertebrae and the three *Hox* genes of paralog group 6 are involved in the development of the cervicothoracic transition. The expression of *HoxD-4* starts at CV1, whereas the anterior expression limits of *HoxB-4* and *B-5* are at CV2 [7, 31, 32]. The expression of *HoxA-4*, *C-4* and *A-5* starts at CV3 [7, 9]. *HoxC-5* is expressed in the posterior part of the neck at CV6 [7]. The anterior expression limits of *HoxA-6*, *B-6* and *C-6* are at TV1 marking the transition from neck to trunk [7, 32, 33]. Thus, there are three units of postatlantal vertebrae in the neck that share the same *Hox* code (Fig. 3).

**Table 3:** References for somitic *Hox* gene expression pattern in the mouse. The literature survey focused on embryonic stages at which somitic *Hox* gene expression limits are thought to be well established and stable during further development

Gene	Embryonic day	Reference
*HoxA-4*	E9-13.5	[7]
*HoxB-4*	E9-13.5	[7]
*HoxC-4*	E9-13.5	[7]
*HoxD-4*	E12.5	[31]
*HoxA-5*	E11	[9]
*HoxB-5*	E12.5-13.5	[32]
*HoxC-5*	E9-13.5	[7]
*HoxA-6*	E11.5-12.5	[33]
*HoxB-6*	E12.5-13.5	[32]
*HoxC-6*	E9-13.5	[7]

The review of loss-of-function mutations in the mouse shows how knock-out of *Hox4* and *Hox5* genes affect the development of the axial skeleton (Table 4).

**Table 4:** Single and multiple loss-of-function homozygote mutations in *Hox4* and *Hox5* paralog group (PG) and effect on the axial skeleton in the mouse, in particular focusing on the cervical vertebral column. Mutations in *Hox3* and *Hox6* PG also affect vertebral development, but are usually confined to the atlanto-occipital and thoracic region [75, 76]. Ref. = references

Gene	Old name^a^	Mutation target	Homeotic transformation	Modifications	Ref.
*Hox4 PG* (excl. *HoxC-4*)	*-*	CV2-5	(partial) anterior transformation	CV2-5 developing C1-type characteristics	[40]
*HoxA-4*	*Hox-1.4*	CV3	partial anterior transformation	CV3 developing CV2-type neural spine	[38, 39]
		CV7	posterior transformation	cervical ribs at CV7	
*HoxB-4*	*Hox-2.6*	CV2	partial anterior transformation	CV2 developing anterior arch and extra ventral tubercula	[77]
*HoxC-4*	*Hox-3.5*	CV7	partial posterior transformation	cervical rib heads on CV7	[78]
		TV3	anterior transformation	prominent spinous process on TV3	
		TV8	anterior transformation	vertebrosternal ribs on TV8	
*HoxD-4*	*Hox-4.2* */-5.1*	CV1-CV3	anterior transformation	fusion of neural arches at CV1-3	[79]
		CV2	anterior transformation	CV2 developing anterior arch, CV1-type lateral articular surfaces	
		CV7	posterior transformation	cervical ribs at CV7	
*Hox5 PG*	*-*	CV3-TV2	anterior transformation	CV3-TV2 developing C2-type characteristics	[63]
		CV7	anterior transformation	CV7 developing CV6-like transverse foramina	
*HoxA-5*	*Hox-1.3*	CV6	anterior transformation	absence of ventral tubercula on CV6	[61, 62]
		CV7	partial posterior transformation	cervical ribs on CV7	
		LV1	anterior transformation	thoracic ribs on LV1	
*HoxB-5*	*Hox-2.1*	CV6	anterior transformation	absence of ventral tubercula on CV6	[32]
		CV7	anterior transformation	CV7 developing CV6-like ventral tubercula, transverse foramina	
		TV1	anterior transformation	absence of ribs on TV1	
		shoulder girdle	rostral shift of shoulder girdle	forelimbs shifted anteriorly	
*HoxC-5*	*Hox-3.4*	-	-	-	-

^a^after Scott MP: A rational nomenclature for vertebrate homeobox (HOX) genes. *Nucleic Acids Res* 1993, 21:1687-1688

## Discussion

### Morphological modularity and vertebral *Hox* code in living mammals

The morphological difference between the atlas (CV1) and the successive vertebrae is evidently distinct and TV1 markedly differs from the cervical vertebrae by the presence of ribs connected to the sternum [34]. For the postatlantal cervical vertebral column of the mouse, the present analysis detected a morphological three-subunit pattern, indicating that two distinct shape changes occur between successive cervical vertebrae (between CV2-3 and between CV5-6). This is consistent with previous 2D studies that investigated the shape changes along the vertebral column of the mouse by means of traditional morphometrics [35] and Fourier outline analysis [15, 36, 37], but extends these investigations by assigning the cervical vertebrae to morphological modules.

Intriguingly, there is a striking correspondence between the anterior expression limits of *Hox* genes and the morphological modularity of the vertebral column in the mouse including the atlas, the axis, three anterior and two posterior cervical vertebrae as distinct vertebral subunits (Fig. 3.). Furthermore, loss-of-function experiments elucidate the direct relation between *Hox* genes and vertebral morphology (Table 4). For instance, the knockdown of *HoxA-4* results in a partial anterior transformation of CV3 because it develops a CV2-type neural spine [38, 39]. The effect of all mutations is confined to the expression area of the respective *Hox* gene. However, the effect of some mutations is restricted particularly to the anterior expression area of the respective *Hox* gene (*Hox4* group), whereas other *Hox* mutations affect more posterior expression areas (*Hox5* group) (Table 4). Since the expression of genes other than the mutant *Hox* gene usually remains unaffected, this highlights the relative contribution of each gene to vertebral development (e.g. [40]).

In summary, the present results show that the morphological pattern is reflected in the *Hox* gene expression pattern not only in living archosaurs [13], but also in the mouse (this study).

### *Hox* genes and the evolution of mammals

#### Numerical constraint

The high level of conservatism in the number of cervical vertebrae in mammals is present early in their evolutionary history significantly predating the origin of crown mammals [41, 42]. Non-mammalian synapsids reflect the conserved axial configuration of extant relatives since they display little variation in cervical count [41, 43, 44]. Furthermore, with a range of five to eight vertebrae in the non-mammalian synapsid neck [41], the variation is within the limits observed for modern mammals (e.g. five to nine cervical vertebrae in sloths [45]). However, it has to be noted that the numerical variation in modern mammals such as sloths is likely to be secondary deviation and that the cervicothoracic transition still occurs between vertebrae 7 and 8 [46–48]. In combination with the conservatism in morphological modularity detected in the neck of different mammalian species (this study, [14–16, 49]), the constraint in cervical count indicates that the *Hox* code responsible for the development of the cervical vertebral column was likely established subsequent to the divergence of the mammalian lineage.

Previous studies reported on how changes in the cervical vertebral column correlate with changes in the *Hox* gene expression pattern in living archosaurs [8, 13]. The present work on mammals provides further evidence for the strong link between the *Hox* code and quantifiable vertebral morphology. Due to the high conservatism in the number of cervical vertebrae, the developmental and morphological modularity detected in the neck of the mouse may serve as a model for the early mammalian neck (Fig. 3). Indeed, there is empirical evidence suggesting that the fixation of cervical count occurred in the Triassic [14, 21]. This may be associated with the similar morphological modularity and, thus, the equivalent *Hox* gene expression pattern as observed in the mouse (Fig. 3).

The detected morphological modularity in the neck of the mouse appears to be a general pattern typical for living mammals with seven cervical vertebrae [14, 15, 37, 50]. This strongly conserved modularity may be explained by a combination of numerical and functional constraints resulting in a evolutionary-developmental trade-off. On one side, the number of cervical vertebrae in mammals is highly conserved and, thus, restricts variation. On the other side, the head-neck system is a vital functional system and displays morphological adaptations to fundamental functional demands, which also limits variation. Fundamental functional specializations include the atlas–axis complex, which is specialized for facilitating mobility of the head, and the cervicothoracic transition, which forms the junction of the highly mobile cervical vertebral column to the relatively stiff thoracic vertebral column [2, 51]. Indeed, the comparative morphological analysis of rat and bat vertebrae revealed that CV2 in rat and bat are very similar, as isCV6 between both species [15]. In contrast, CV3–5 are morphologically very characteristic of each species [15].

#### Atlas-axis complex (CV1-2) and *HoxD-4*

In mammals, the cervical vertebral column typically forms a S-shaped curvature with one flexure in the anterior region (CV1–3) and the other in the posterior region (CV6–TV1) [2]. At rest, the cervical vertebral column of quadrupedal mammals is maximally flexed at the craniocervical transition [51, 52]. In order to facilitate mobility in the anterior cervical region, the first two vertebrae (CV1–2) display almost universally, highly specialized conditions [1, 53]. This functional specialization is closely linked to the evolution of a distinct neck by the separation of the pectoral girdle from the skull and, thus, already present early in the evolutionary history of tetrapods [53]. The plesiomorphic state is that the individual elements of the two first cervical vertebrae (neural arches, centra, and intercentra) remain discrete [43, 53]. Only in mammals, the atlas becomes a ring-shaped structure solely formed by the neural arch and the intercentrum of the first cervical vertebra, and the axis develops an anterior extension (odontoid process) by incorporation of the centrum of CV1 [43, 54–56]. Although non-mammalian synapsids already possessed a typical ring-shaped atlas and a odontoid-like structure, the later is first not fused to CV2 [57]. The fusion of the odontoid process to CV2 as seen in modern mammals occurs sometime in the Mesozoic since cynodonts display the odontoid as a fused structure [43].

In mouse, the morphological differentiation of the atlas–axis complex is mediated by the expression of *HoxD-4* which starts at the CV1, and that of *HoxB-4* and *HoxB-5* which both start at CV2 (Fig. 3). In archosaurs (represented by chicken and crocodilian), however, only the anterior expression limit of *HoxB-5* is at CV2, whereas *HoxD-4* shares its anterior expression limit with *HoxB-4* at CV3 [7–9, 13]. Since archosaurs do not develop the high degree of specialization of the atlas–axis complex seen in mammals, this may indicate that the shift of *HoxB-4* and *HoxD-4* expression likely occurred in Mammaliformes resembling the pattern of the mouse (Fig. 3). Strikingly, *HoxD-4* mutations affect the development of CV1-3 (Table 4).

#### Posterior unit (CV6-7) and *HoxC-5*

The base of the neck is the second cervical vertebral region of high mobility, particularly in dorsoventral direction [2, 51]. Typically, it is markedly extended at resting position contributing to the lordotic curvature of the cervical vertebral column [51, 52]. In both archosaurs and mammals, the anterior expression limit of *HoxC-5* is at the penultimate cervical vertebra [7, 8, 13], irrespective of the cervical count (Fig. 3). This indicates that the association between *HoxC-5* and the posterior cervical vertebrae is established early during the evolution of a distinct neck in tetrapods and, thus, a posterior expression of *HoxC-5* is likely present in synapsids as well. The same appears to be true for the expression of the *Hox6* PG genes, which marks the cervicothoracic transition in species with varying numbers of cervical vertebrae.

The effect of a single loss-of-function mutation in *HoxC-5* on the axial skeleton can not be evaluated since it has not been studied to date. However, triple *Hox5* mutations affect the development of cervical and anterior thoracic vertebral column (Table 4).

#### Cervical ribs and *HoxA-5*

In contrast to the rudimentary, fused cervical ribs in modern mammals [58], most Mesozoic mammals possessed freely articulating cervical ribs [14, 42, 59]. This suggests differences in the genetic code because *Hox* genes are involved in the specification of rib-bearing and rib-free vertebrae. For instance, the *Hox 10* group specifies the rib-less lumbar vertebral region [60]. *Hox10* loss-of-function mutants (disruption of all three paralogous genes) completely lack lumbar vertebrae, but display the entire lumbosacral region homeotically transformed to a thoracic-like morphology including ectopic ribs [60]. With regard to the neck, *HoxA-5* is involved in mediating the development and suppression of cervical ribs [9, 12, 13, 61, 62]. Single mutation experiments in the mouse revealed that disruption of *HoxA-5* results in homeotic transformations of the axial skeleton confined between CV3 and TV2 (Table 4, Fig. 3a) and one of the most frequent morphological modifications is the development of a pair of ribs on CV7 [61–63]. Additionally, comparative analyses with living non-mammalian tetrapods provide support for the rib-promoting and rib-suppressing role of *HoxA-5* [8, 9, 13]. Alligator and crocodile possess free cervical ribs and the expression limit of *HoxA-5* starts in the posterior cervical vertebral column [8, 9, 13] which contrasts with the more anterior expression limit of *HoxA-5* observed in the mouse (Fig. 3). Therefore, it is possible that the *Hox* code in the neck of non-mammalian synapsids and early mammals includes a posterior expression of *HoxA-5*. Given the loss of free ribs on the cervical vertebrae (by reduction and fusion), which occurred in the Cretaceous [14, 42, 59], the anterior expression limit of *HoxA-5* may be shifted in Mammaliformes resembling the pattern of the mouse (Fig. 3).

However, it should be noted that the *HoxA-5* loss-of-function mutants did not develop ribs in the entire cervical vertebral column and still retained rib-free cervical vertebrae (CV3 to CV6) [61–63]. This leaves the question open if other *Hox* genes share some function of *HoxA-5* and, thus, may compensate for the *HoxA-5* vertebral phenotype. To date, we lack information in terms of the cervical vertebral column, but multiple mutants in the *Hox5* group revealed partial functional redundancy among the *Hox* genes in the other parts of the animal body, such as the lung [64, 65] and the forelimb [66]. For instance, mice with mutations of the *HoxA-4* gene [38], the *HoxA-5* gene [61] and the *HoxA-6* [39] display a overlapping phenotype (cervical ribs on CV7). Furthermore, overexpression of *HoxA-4* results in suppression of rib formation at CV7 [38]. However, this is not necessarily an argument against the postulated posterior expression of *HoxA-5* in non-mammalian synapsids and early mammals, but emphasizes the combinatorial nature of *Hox* gene activity in axial patterning.

Conclusive evidence for the *HoxA-5* hypothesis in the mammalian ancestor, however, may be drawn from future analyses on the *Hox* gene expression pattern in monotremes, which are among the only living mammals that retained cervical ribs [67]. Indeed, except for some differences in the *HoxC* cluster, monotremes appear to have the identical *Hox* gene inventory as marsupials and placental mammals [19], but to date, there is no information on the pattern of gene expression. Intriguingly, the ossification of cervical ribs occurs late (long after the thoracic ribs) in monotremes, which is in contrast to non-mammalian tetrapods [68]. Thus, it has been suggested that the occurrence of this delay in monotremes is linked to developmental changes involved in the disappearance of cervical ribs and that these changes could have been already established in the mammalian ancestor [68].

A parallel case observed for birds may provide further support for the evolutionary *HoxA-5* hypothesis. In birds, whose cervical ribs are present, but fused to the vertebrae, the cervical ribs ossify late as in monotremes [68, 69]. In contrast to mammals and crocodilians, the anterior expression limit of *HoxA-5* is in the middle region of the neck in the chicken [9] and *HoxA5* knockdown results in defects of the cervical ribs [70].

Altogether, the functional link between *HoxA-5* and cervical ribs is evident and, thus, it is reasonable to conclude that a shift in the expression pattern of *HoxA-5* occurred at some point during the evolution of mammals. However, more studies in non-model organisms are required to further elucidate the evolutionary role of *HoxA-5* in axial patterning.

## Conclusions

In contrast to non-mammalian tetrapods, in which the variation in vertebral count also plays a central role, neck kinematics in mammals are almost exclusively related to interspecific variation in vertebral morphology [2, 51, 71] because the number of cervical vertebrae is highly constrained (virtually always seven CV) [22, 23]. For instance, longer vertebrae and flexible intervertebral joints generally allow for a higher degree of neck mobility [23, 72], whereas shorter vertebrae and fusion or additional processes between vertebrae increase stiffness and provide a firm and unyielding support to the skull [23, 73, 74]. Despite these significant morphofunctional differences, the pattern of shape change within the neck appears to be consistent among diverse mammal taxa [this study, 14, 15, 48, 58]. On basis of the correlation between *Hox* code and vertebral morphology, the modularity detected in the neck of the mouse is a reasonable model for mammals with seven cervical vertebrae. Morphological differences in the pattern of shape change within the neck between modern mammals and non-mammalian synapsids indicate that modifications in the *Hox* code likely occurred during the evolution of synapsids, including an anterior shift in *HoxA-5* expression in relation to the reduction of cervical ribs and an anterior shift in *HoxD-4* expression linked to the development of the highly differentiated atlas-axis complex. Thus, the present study provides a reliable basis for further research on the evolution of the vertebral column in mammals including future morphological analyses in fossils as well as *Hox* gene expression studies in non-model mammalian species.
